# Effects of Annealing on the Radio Frequency Sputtered CuO/ZnO Thin Film Heterostructure for Optoelectronic Applications

**DOI:** 10.3390/ma19040789

**Published:** 2026-02-18

**Authors:** Sinthamani Sivaprakasam, Sudhakar Bharatan, Ranjithkumar Mohanam, Sudharsanam Subramaniyam

**Affiliations:** Department of Electrical and Electronics Engineering, Sri Venkateswara College of Engineering, Sriperumbudur 602 117, India; sinthamani@svce.ac.in (S.S.); mranjith@svce.ac.in (R.M.); sudharsanam@svce.ac.in (S.S.)

**Keywords:** RF sputtering, thin film solar cell, MoS_2_, FTO, x-ray diffraction, raman spectroscopy, photoluminescence

## Abstract

**Highlights:**

**What are the main findings?**

- ZnO and CuO thin films prepared by RF sputtering were optimized under varying annealing temperatures and ambient conditions, leading to improved structural quality.
- Annealed ZnO films showed increased grain size and strong sub-band absorption, with a PL band-edge at 3.27 eV.
- Annealed CuO films exhibited direct band-to-band absorption at 2.89 eV due to the disappearance of defect-related sub-bands.
- Raman spectroscopy and XRD analysis confirmed reduced defects and enhanced crystallinity in annealed CuO films.
- Al/FTO/MoS_2_/CuO/ZnO/Al heterostructure demonstrated improved photocurrents, achieving ~5 mA in the dark and ~9 mA under illumination.

**What are the implications of the main findings?**

- The ZnO/CuO heterostructure device shows improved photocurrent response with the insertion of MoS_2_ as a hole transport layer, suggesting a viable pathway for high-performance heterostructure devices.

**Abstract:**

ZnO and CuO thin films were deposited separately using the radio frequency (RF) sputtering technique, and the effect of annealing in nitrogen and oxygen ambient environments was investigated. In this article, structural, optical, vibrational, and electrical characterizations were sequentially performed using techniques such as X-ray diffraction (XRD), UV–visible spectroscopy (UV-vis), Raman spectroscopy, photoluminescence (PL) spectroscopy, and current-voltage measurements using a DC four-probe station. XRD confirmed a high-crystallinity and wurtzite structure for ZnO, with the preferred orientation being along the c-axis (0001), and a monoclinic structure for CuO, with preferential orientation along the (002) axis. The absorption edges of the ZnO and CuO thin films were determined to be 3.24 eV and 2.89 eV, respectively. However, Urbach tails were observed only in the ZnO thin films, confirming the presence of localized Zn interstitials and oxygen vacancies. The absorption of CuO showed weak Urbach tails, suggesting that the defects were not localized. Raman spectroscopy performed on the ZnO and CuO thin films showed the appearance of weak E_2_(high) and prominent A_g_/B_2g_ modes, confirming the presence of ZnO and CuO bonding states, respectively. PL studies revealed room temperature emission for both the CuO and ZnO thin films, which is crucial for thin film solar cells and photodetectors. Two thin film heterostructures were fabricated with and without MoS_2_ (a hole transport layer) on FTO substrates. The Al/FTO/CuO/ZnO/Al heterostructure revealed a rectifying behavior with a photo current of 2 mA in the dark, whereas light-induced characteristics resulted in a photocurrent of 5 mA. The Al/FTO/MoS_2_/CuO/ZnO/Al heterostructure exhibited a similar rectifying behavior, with improved photo currents of 5 mA in the dark and 9 mA in the light.

## 1. Introduction

ZnO and CuO are some of the most extensively studied oxide semiconductors. The availability of these materials, being abundant in nature, and their non-toxicity make them effective materials for photodetectors [1] and thin film solar cells [2]. Numerous experiments have been carried out on various metal-doped oxides of ZnO (Al, Ga, Mg) and CuO (Co, Zn, Ni) [3,4,5,6,7,8] due to their high transparency and high absorbance in the visible-light region, respectively. CuO is predominantly a p-type semiconductor with a band gap ranging between 1.2 and 1.8 eV for the cupric phase monoclinic structure. This makes CuO a promising near-IR absorption layer for solar cell applications. It is well known that copper oxide exists in two distinct forms, such as copper (II) oxide (CuO, tenorite) and copper(I) oxide (Cu_2_O, cuprite) [9]. CuO, being a p-type semiconductor, exhibits a monoclinic structure with a narrow bandgap, high absorption, and weak thermal properties. The low thermal conductivity of CuO is known to reduce heat-related losses, improve stability, and enhance long-term performance. CuO also exhibits a high absorption coefficient of the order of >10^5^ in the visible region (400–800 nm). This enables the efficient absorption of photons across most of the visible spectrum, making it attractive as a solar absorber compared to conventional semiconductor materials, such as silicon, which has a relatively lower absorption coefficient of the order of 10^4^ cm^−1^. Alternatively, Cu_2_O forms a cubic structure and exhibits a wider bandgap of 1.8–2.5 eV [10]. Due to the above merits and the ease of its fabrication, CuO films are potential candidate for applications in photodetectors, sensors, and solar cells.

ZnO exhibits excellent electrical and optical properties (3.3 eV bandgap), with higher mobility, strong optical transparency, and bandgap tunability. The role of ZnO in the p-n heterostructure is that it provides not only optical transparency but also forms an effective electron transport layer. Although the efficiencies of metal–oxide thin film solar cells are lower than those of perovskite solar cells (26.7% efficiency) [11], metal–oxides play a vital role in improving solar-cell efficiency and chemical stability.

The combination of CuO and ZnO forms a p-n heterostructure and has been realized in a variety of applications, such as solar cells [12], sensors [13], optoelectronics [14,15], and biomedical engineering [16]. Traditional fabrication techniques, such as molecular beam epitaxy (MBE) [17], pulsed laser deposition [18], metal–organic chemical vapor deposition [19], sol–gel [20], and radio frequency (RF) magnetron sputtering [21], have been employed in fabricating devices such as photodetectors, solar cells, and photocatalysts. Among these techniques, RF sputtering is one of the most preferred, which offers scalability, uniformity, low-temperature growth, and a low-cost option for oxide semiconductors.

Even though RF-sputtered ZnO thin films are known to exhibit high mobilities, they are dominated by native defects arising from high background carrier concentrations, which can be detrimental to device performance [14,15,16,17]. Defect densities in ZnO are known to modify the optical and electrical properties crucial for various optoelectronic applications. However, RF sputtering employs a non-equilibrium growth technique that helps control defect densities in thin films by modifying deposition parameters [22,23,24]. Some defects, including oxygen vacancies and Zn/Cu interstitials, depend on gas partial pressures, deposition conditions, and annealing conditions [25]. In this work, changes in the annealing conditions will be characterized by various techniques, such as XRD, Raman spectroscopy, SEM, UV–visible spectroscopy, and photoluminescence, and their impact on device performance will be evaluated by a DC electrical characterization setup.

The use of metal–oxide thin films allows us to design visible-light-absorbing active layers for the efficient transportation of carriers due to minimum interface defect states [26]. Due to these capabilities, CuO was selected as the solar absorber, coupled with n-ZnO. Many groups have reported much lower power conversion efficiencies of 1–2% for the above combination of CuO/ZnO [27]. Kaphle et al. have reported the highest efficiency, at 2.11% for CuO/Co-doped ZnO with a MoO_3_ buffer-layered solar cell, to the best of our knowledge [28]. However, a solar cell efficiency of 30% was theoretically reported as being obtained using a CuO/ZnO-based heterostructure. The reasons for these low power conversion efficiencies are due to unintentional defects, such as vacancies and interstitials, present in the layers. Similarly, CuO thin film photodetectors have been extensively studied because of their narrow bandgap and low-cost deposition technique, despite the challenge of defect states [5]. Annealing is a major process step in controlling these defects. Hence, our work focuses on the reasons for the drop in efficiency and on ways to improve it by changing post-deposition annealing methods under various ambient conditions. The desirable properties of thin films were obtained by optimizing the variation in deposition parameters such as RF power, gas flow, annealing temperature, and ambient. In this work, an Al/FTO/CuO/ZnO/Al thin film heterostructure was fabricated and electrically characterized. In addition, MoS_2_ was used as a hole-transport layer to study the improvement in the photo current. Based on thin film optimization, thin film heterostructures were fabricated, and their electrical properties were examined.

## 2. Materials and Methods

### 2.1. ZnO and CuO Thin Films

Metal–oxide thin films were deposited on p-Si substrates (5–10 Ω.cm resistivity) using RF magnetron sputtering. The substrates were cleaned using electronic grade chemicals (Thermo Fisher Scientific, Waltham, MA, USA) starting with RCA1 (Radio Corporation of America), RCA2, and HF dip to get rid of surface impurities [29]. The Radio Corporation of America developed a unique cleaning method for silicon substrate to remove organic and ionic contamination from the surface. RCA1 cleaning was carried out to remove organic compounds using a solution mixture of DI water: NH_4_OH: H_2_O_2_, in the ratio of 5:1:1 and heat treatment at 75 °C for 10 min. RCA2 cleaning was subsequently carried out using a 6:1:1 mixture of DI water: HCl: H_2_O_2_ and heat treatment at 75 °C for 10 min. After RCA2, the substrate was dipped into hydrofluoric acid for 5 s to remove native oxide [29].

First the chamber was pumped down to 5 × 10^−5^ mbar, and the RF power was set for thin films as per the data given in Table 1, with an argon:oxygen flow rate ratio of 2:1. Aalborg mass flow controllers (Aalborg Instruments and Controls Inc., Orangeburg, NY, USA) were used for the controlled argon and oxygen gas flow. During both the ZnO and CuO depositions, the target-to-substrate distance was maintained at 7.5 cm. The targets were pre-sputtered for 10 min to obtain homogeneous thin films. A series of ZnO and CuO films was deposited at room temperature and 150 °C, respectively, as shown in Table 1, and optimization of thin films was carried out.

The ZnO thin films of 180 nm thick were deposited using a 99.99% pure ZnO target of 2″ diameter. Ex situ annealing was carried out on the samples SZ2 in the presence of nitrogen in the tubular chamber. CuO thin film was deposited on a silicon substrate (SC1) for 45 min with a substrate temperature of 150 °C, and SC2 was annealed (in situ inside the sputtering chamber) in the presence of oxygen for one hour at 300 °C using a Cu target. Two-inch diameter, 3 mm thick, and 99.99% pure sputtering targets of ZnO, Cu, and MoS_2_ were purchased from Ultrahigh Vacuum Solutions LLP, Bangalore, India.

Theta/2 Theta X-ray diffraction was carried out on Samples SZ1, SZ2, SC1, and SC2 using a Rigaku Smart Lab X-Ray Diffractometer (Rigaku Corporation, Tokyo, Japan). Subsequently, various thin film parameters, such as crystallite size and strain, were calculated from the XRD patterns using peak intensities and the full width at half maximum (FWHM). Transmission and absorbance spectra were obtained for Samples SZ1, SZ2, SC1, and SC2 using a UV-1650PC Shimadzu spectrophotometer (Shimadzu Corporation, Kyoto, Japan) in the visible region 300–800 nm. A slit width of 1–5 nm, a step size of 1 nm, and a Photomultiplier Tube R6872 (PMT) (Shimadzu Corporation, Kyoto, Japan) was employed as a detector for the measurements. Raman spectroscopy was carried out using a LabRam HR Raman spectrometer (Horiba Jobin Vyon, Kyoto, Japan). The Raman spectra were obtained in a backscattering geometry using the 532 nm solid-state laser as the excitation source and liquid nitrogen-cooled Si as the detector. The power at the sample was 3 mW, the diffraction grating was 1200 with l/mm, and the confocal aperture was set at 100 μm with an accumulation time of 45 s. Room-temperature photoluminescence was performed using a 266 nm deep ultraviolet (DUV) laser as the excitation source and a Si photodetector. The scanning electron microscopy (SEM) images were obtained using a Zeiss Ultra55 scanning electron microscope with an accelerating voltage of 2 KV (Zeiss, Jena, Germany).

### 2.2. Thin Film Heterostructures

**Figure 1:**
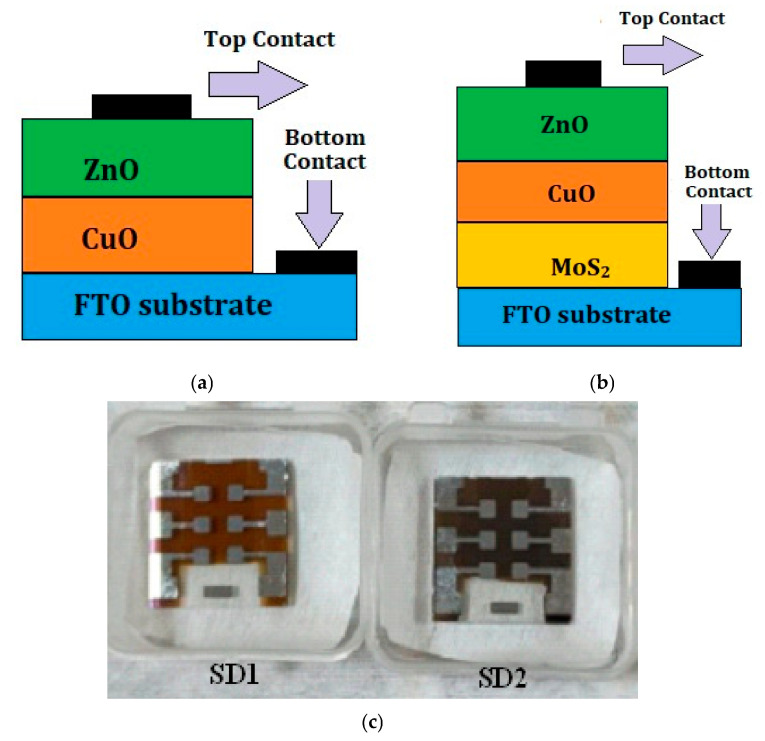
(**a**) Structure (SD1); (**b**) structure (SD2); (**c**) photographs of devices SD1 and SD2.

We also fabricated Al/FTO/CuO/ZnO/Al and Al/FTO/MoS_2_/CuO/ZnO/Al thin film heterostructures, as shown in Figure 1a,b, using a combination of RF sputtering and thermal evaporation on fluorine-doped tin oxide (FTO) glass substrate, as shown in Table 2. The heterostructure layers were sequentially deposited in vacuum using individual Cu and ZnO targets. First, 180 nm of CuO thin film was deposited, followed by in situ annealing at 300 °C in an oxygen ambient, inside the sputtering chamber. This is followed by the deposition of a 50 nm thick ZnO layer and external annealing in a tubular furnace at 350 °C for 1 h in a nitrogen ambient. Both the top (Al) and bottom contacts (Al) were deposited using thermal evaporation. Final contact annealing was carried out externally at 350 °C for 15 min. The I–V characteristics of fabricated devices shown in Figure 1c were later investigated using the Cascade Summit 11000B-M probe station (Cascade Microtech, Beaverton, OR, USA).

## 3. Results and Discussion

### 3.1. X-Ray Diffraction

#### 3.1.1. XRD of ZnO Thin Films

**Figure 2:**
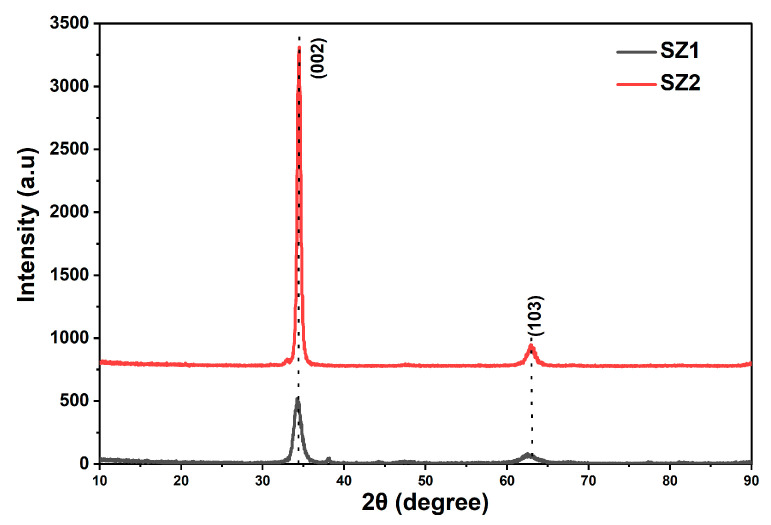
XRD pattern of ZnO thin films SZ1 and SZ2.

Figure 2 represents the XRD pattern of ZnO samples SZ1 and SZ2, deposited at room temperature (RT) and annealed at 350 °C, respectively. Both SZ1 and SZ2 exhibit dominant (002) XRD peaks at 34.3° and 34.4°, respectively, which comply with the data of JCPDS card No. 36-1451 [30,31,32]. Annealing resulted in a 5× increase in the (002) peak, attesting to improved crystalline quality. In addition to the dominant (002) peak, a weak (103) XRD peak was observed in both SZ1 and SZ2 samples, indicative of a polycrystalline ZnO wurtzite structure.

Based on the FWHM values of the (002) XRD peak, the mean crystallite size of the samples was calculated. Narrower XRD peaks typically indicate larger crystallite size. From the (002) XRD FWHM data, various parameters, such as grain size (*D*), lattice strain (δ) and dislocation density (ε), were calculated using Debye–Scherrer’s formula [33,34,35] and are presented in Table 3.

(1) $$D=\frac{0.9\lambda }{\beta cos\theta }\;$$

(2) $$\varepsilon =\frac{1}{D^{2}}$$

(3) $$\;\delta =\frac{\beta }{4tan\theta }$$

Sample SZ2 exhibits a narrow FWHM of 0.451°, which corresponds to a larger crystallite size of 19 nm, whereas Sample SZ1 shows a broader FWHM of 0.945°, which corresponds to a smaller crystallite size of 9.11 nm. Even though a larger grain size attests to improved crystalline properties, the presence of the (103) XRD peak suggests that ZnO does not only have c-axis orientation. Insights into the ZnO texture will be obtained in the later sections through SEM image analyses. While both the samples exhibit weak (103) peaks, the higher (002)/(103) intensity ratio of 25 in the annealed ZnO thin film, when compared with the ratio of 10 in the unannealed thin film, suggests stronger c-axis orientation, which attests to the good quality of the annealed one (SZ2).

#### 3.1.2. XRD of CuO Thin Film

**Figure 3:**
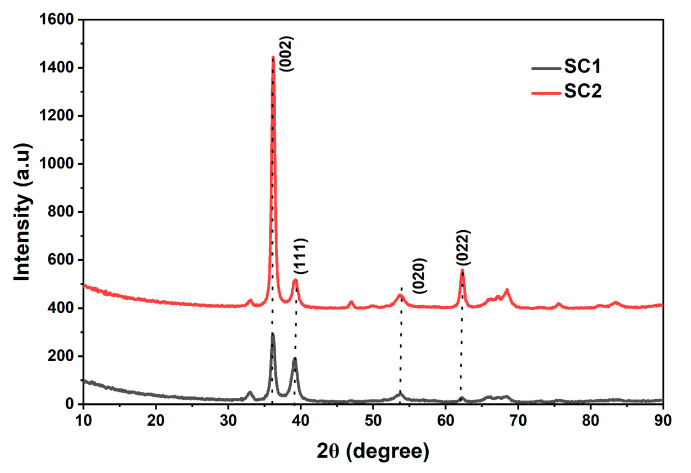
XRD pattern of CuO thin films SC1 and SC2.

Figure 3 represents the XRD pattern of CuO thin film samples, SC1 and SC2, both deposited at 150 °C, and Sample SC2 alone, which was subsequently annealed at 300 °C in O_2_ ambient. Both CuO thin films exhibit a dominant peak at 35.4° corresponding to the CuO (002) plane. All the diffraction peaks are consistent with the standard data (JCPDS card no. 89-2531) and attest to the formation of a monoclinic structure of CuO [36]. Sample SC1 exhibits the characteristic monoclinic CuO peak at 36.8° (002) and 39° (111). A strong (111) CuO peak indicates good crystalline quality. Annealed sample SC2 exhibits a significant improvement in (002) XRD peak, in addition to the appearance of a symmetrical (022) XRD peak at 62°. The appearance of a strong (022) peak suggests improved crystalline quality and stabilization of the monoclinic CuO phase after annealing. The symmetry of the (002) and (022) XRD peaks is indicative of reduced strain and fewer defects in the annealed CuO layers. A high (002)/(111) intensity ratio of 10 was achieved in the annealed sample as compared to an intensity ratio of 5 in the unannealed sample (SC1), suggesting the preferred orientation is along the (002) plane.

Similar to ZnO thin films, various grain parameters were calculated for CuO films as well, using Debye–Scherrer’s formula [37,38] and are presented in Table 4. As expected, annealing resulted in a reduction in the lattice strain in annealed sample SC2.

### 3.2. Raman Characteristics of ZnO and CuO Thin Films

#### 3.2.1. Raman Characteristics of ZnO Thin Film

**Figure 4:**
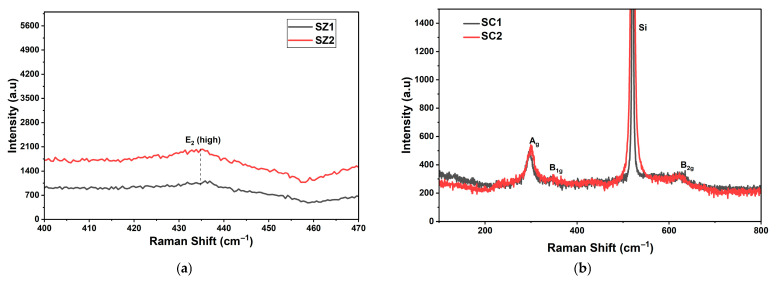
(**a**) Raman spectrum of ZnO thin films SZ1 and SZ2. (**b**) Raman spectrum of CuO thin films.

Figure 4a shows the Raman spectra of ZnO thin films SZ1 and SZ2. Figure S1 shows the Raman spectra of ZnO thin films, SZ1 and SZ2, with silicon peaks. Both samples exhibit E_2_ (high) mode, characteristic of a ZnO wurtzite structure. A typical E_2_ (high) mode observed at 437 cm^−1^ for the wurtzite ZnO structure appears at redshifted 435 cm^−1^ in both ZnO films. The slight redshift implies the presence of lattice distortion, possibly due to defects related to crystallite boundary stress and/or oxygen vacancies in the ZnO layers. Annealing results in a slight increase in the E_2_ (high) peak, which could be attributed to the reduction in point defects. However, the asymmetricity of the peak suggests that the defects are not completely annihilated. There have been reports [39] of defect formation, such as tensile strain and oxygen vacancies, due to the bombardment of energetic ions while sputtering, which could explain the redshifted E_2_ (high) peak.

#### 3.2.2. Raman Characteristics of CuO Thin Films

Figure 4b shows the Raman spectra of CuO samples with two distinct Raman peaks at 296–300 cm^−1^, corresponding to A_g_ mode, and 624–630 cm^−1^, corresponding to the B_2g_ modes of CuO. The strong peak at 520 cm^−1^ corresponds to the Si substrate. A typical Raman spectroscopy is known to exhibit three modes, Ag, B_1g_, and B_2g_, of the crystal structure of monoclinic CuO [40]. Marked improvements in the peak intensities of Ag and B_2g_ modes are observed in the annealed CuO sample, which is indicative of better CuO bonding. As-deposited and unannealed sample SC1 exhibits a redshifted Ag peak at 295 cm^−1^, and a blueshifted B_2g_ peak at 629 cm^−1^. The redshifted Ag peak at 295 cm^−1^ in Sample SC1 is likely due to the sputter-induced defects, due to lattice distortion, and non-stoichiometry of CuO alloy [41]. Upon annealing, the improvement in layer quality is clearly evident due to the blue shift in Ag mode to 300 cm^−1^, which is close to the typical vibrational Ag mode for monoclinic CuO thin film. The decrease in stress is reflected in the corresponding strain, calculated using XRD data (Table 4).

With regard to the second characteristic, the B_1g_ peak related to O-related vibrations, only the sample SC2 exhibits a weak hump at 346 cm^−1^. The presence of the B_2g_ CuO peaks in the range of 625 to 629 cm^−1^ in both samples is due to the lattice-bending vibrations in the monoclinic tenorite CuO [40,41]. Both samples were deposited at 150 °C and cooled down to RT, which could give rise to lattice contraction resulting in compressive strain. [42]. This explains the blueshift of B_1g_ peak relative to the normally observed B_1g_ peak near 612–615 cm^−1^ in unstrained CuO films. It should be noted that other forms of CuO bonds, such as Cu_2_O and Cu_4_O_3_, were not observed in both samples. Raman bands at around 218 (corresponding to Cu_2_O) and 533 cm^−1^ (corresponding to Cu_4_O_3_) were not observed [43]. The increase in peak intensity reveals the increase in the bonding strengths of CuO when deposited at 150 °C, followed by annealing at 300 °C [44].

### 3.3. SEM

**Figure 5:**
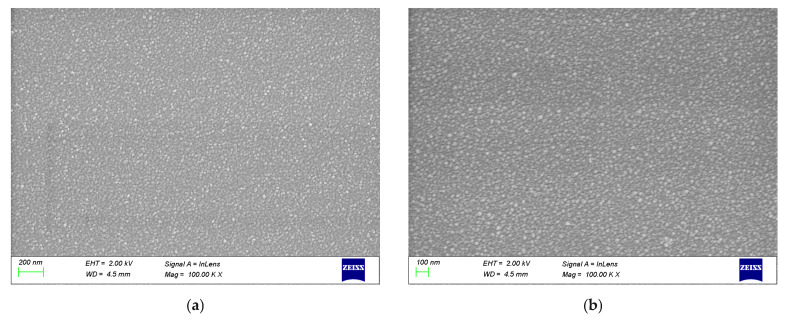
SEM image of ZnO thin films (**a**) SZ1, (**b**) SZ2.

Figure 5a,b presents the SEM images of ZnO samples SZ1 and SZ2. Sample SZ1, deposited at room temperature, exhibits a fine-grained morphology with comparatively smaller particle sizes. In contrast, Sample SZ2 displays distinct contours and larger grains. The grain sizes estimated from XRD analysis are 9.11 nm for SZ1 and 19.27 nm for SZ2, which correspond well with the SEM observations, confirming the presence of uniformly distributed, larger grains in SZ2. SEM images of ZnO sample SZ2 with greater magnification are shown in Figure S2. These findings clearly demonstrate that the crystal size of ZnO is strongly influenced by the annealing process.

### 3.4. UV-VIS–Spectroscopy

#### 3.4.1. UV-VIS–Spectroscopy of ZnO Thin Films

**Figure 6:**
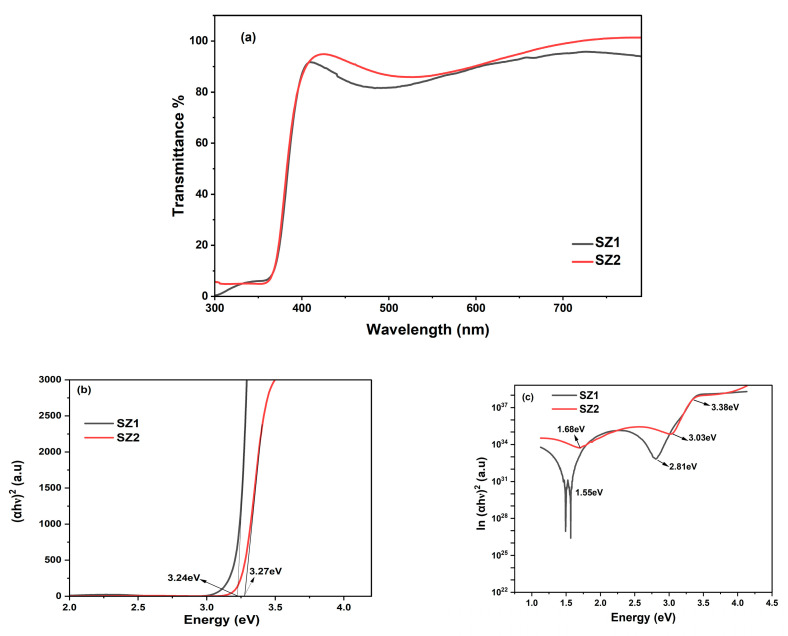
(**a**) Transmittance spectra of ZnO; (**b**) absorption spectra of ZnO; (**c**) ln α vs. energy (eV).

Figure 6a depicts the transmittance plot of as-grown and annealed ZnO thin films, SZ1 and SZ2. The ZnO-CuO-based heterostructure for optoelectronics application requires the combination of good transmittance of ZnO and strong absorption of CuO in the 1.2 eV region. The optimized process parameters for ZnO and CuO thin films were later implemented in the fabrication of the ZnO-CuO thin film heterostructure. Both ZnO thin films exhibit a transmittance of ~85% in the visible region from (350–380 to 750–780 nm), which is transparent enough for solar absorption by the underlying CuO absorption layer. It was observed that Sample SZ1 has a maximum transmittance of 89.96% at 424 nm, and Sample SZ2 reaches a maximum transmittance of 99.05% at 442 nm. UV–visible results indicate a bulk-like absorption for both samples SZ1 and SZ2. Figure 6b compares the absorption edge of both samples. Sample SZ1 exhibits an absorption edge at 3.24 eV, whereas annealing in SZ2 resulted in a blueshift in the absorption edge at 3.27 eV. The relatively low Moss Burstein shift of 0.06 eV could be attributed to the presence of native defects [44]. Sputtered ZnO thin films are known to exhibit point defects, such as Zn interstitials and O_2_ vacancies, contributing to a possible increase in the carrier concentration and explaining the blueshift observed in Sample SZ2.

The effects of point defects on our ZnO samples were analyzed based on the detailed electronic transitions from the UV–visible absorption spectra. The absorption data were delineated into various transitions, such as interband transition in ZnO material, localized band tail transitions (commonly referred to as Urbach tails), and free carrier absorption [45]. Various groups have studied the occurrence of Urbach tails in their ZnO thin films [46,47].

To identify various sub-bands of our samples, a natural log of the absorption edge and the bandgap was plotted. Figure 6c shows the plot between the ln(α) and the bandgap for Samples SZ1 and SZ2. Sample SZ1 exhibits Urbach tails at 1.55 eV and 2.81 eV, which are attributed to the presence of both O_2_ vacancies and Zn interstitials, thereby leading to the creation of deep donor and acceptor levels within the bandgap [25]. In contrast, the annealed sample exhibits weak tails at 1.68 eV and 3.03 eV. The Urbach tails observed in Sample SZ1 are attributed to the presence of the above defects, which are annihilated in Sample SZ2. These defects create deep donor and acceptor bands within the bandgap, leading to strong sub-band absorption. Similar Zn interstitials below the conduction band and O_2_ vacancies above the valence band, with absorptions at 2.8 eV and 1.52 eV, respectively, were observed by Sasikala et al. The fast roll-off at 1.55 eV in SZ1 could be attributed to the presence of highly localized defects. The fast roll-off at 1.55 eV becomes weak in the annealed sample, SZ2, which could be attributed to the reduction in localized defects, particularly Zn interstitials.

#### 3.4.2. UV-VIS–Spectroscopy of CuO Thin Films

**Figure 7:**
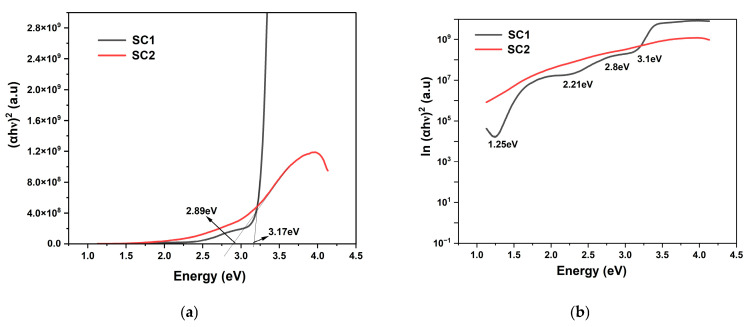
(**a**) Absorption spectra of CuO thin film; (**b**) ln α vs. energy (eV).

Similar Tauc plots between (αhν)^2^ and energy (eV) were plotted to determine the absorption edge of the CuO-based thin films, SC1 and SC2 (Figure 7a). Both the samples show an absorption edge comparable to the bulk material, confirming good-quality CuO thin films. Sample SC1 shows an absorption edge at 3.17 eV (391 nm) with relatively strong absorption in the violet edge of the visible spectrum, whereas annealed Sample SC2 exhibits a redshift in the bandgap with an absorption edge at 2.89 eV, in the blue region of the visible spectrum, conforming with the increase in grain size, as observed in the XRD calculations (Table 4). The observed redshift after annealing can be attributed to a phase transition from Cu_2_O to CuO (or a reduction in mixed-phase Cu_2_O/CuO content), wherein oxidation during annealing promotes the formation of CuO with a lower effective optical transition energy, thereby shifting the absorption edge toward longer wavelengths [47].

The delineation of the sub-bands within the bandgap of Sample SC1 is confirmed from the absorption edge plot in the natural log versus the bandgap. Figure 7b shows the plot between ln(α) and bandgap for Samples SC1 and SC2. Unannealed Sample SC1 clearly shows multiple weak Urbach tails at 1.25 eV, 2.21 eV, 2.8 eV, and 3.1 eV. However, the annealing at 300 °C in Sample SC2 resulted in the disappearance of sub-bands, which could be the result of annihilation of the defects resulting in direct band-to-band absorption at 2.89 eV. The sharp roll-off in ZnO thin films (Figure 6c) represents the presence of highly localized defects, whereas the weak roll-off in CuO thin films suggests that shallow defects only occur in Sample SC1 (Figure 6b) [48].

### 3.5. Photoluminance of ZnO and CuO Thin Films

#### 3.5.1. Photoluminance of ZnO Thin Films

**Figure 8:**
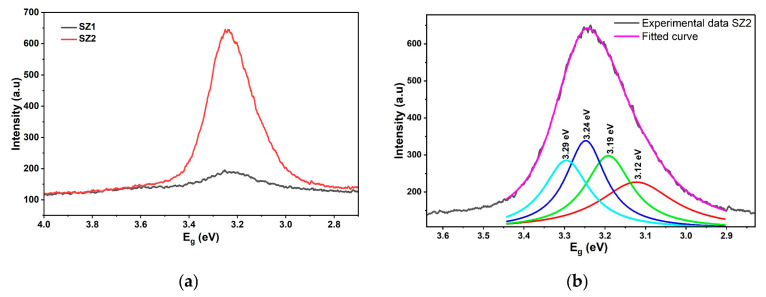
(**a**) Photoluminescence of ZnO thin films SZ1 and SZ2. (**b**) Peak fit of ZnO thin film (SZ2).

Room-temperature photoluminescence of ZnO thin films SZ1 and SZ2 is shown in Figure 8a. Both the thin films exhibit PL peak wavelength at 383 nm, which matches well with the absorption edge of 3.27 eV (SZ1) and 3.24 eV (SZ2) from UV–visible spectroscopy data (Figure 6b) [49]. Annealing resulted in a clear increase in the PL intensity for Sample SZ2 as compared to Sample SZ1, conforming with the XRD and UV–visible spectroscopy data. XRD and UV–visible data confirmed a larger grain size and a low level of defects, respectively, attesting to the enhanced layer quality of Sample SZ2. However, the asymmetrical PL indicates the presence of defects and is validated by the delineation of the broad PL peak into various sub-peaks. It is well known that the energy of typical defect levels is in the range of 3.1–3.49 eV. The choice of the number of bands and their corresponding energies was not arbitrary but guided by both experimental features and the established literature. Specifically, the shoulders and intensity variations observed in the PL spectra indicated the presence of multiple emission centers, which will be described in detail in the following section.

Figure 8b shows the Lorentzian fit of the PL spectrum for the annealed ZnO sample SZ2. The broad PL peak resolved into four sub-peaks with emissions at 3.3 eV, 3.24 eV, 3.2 eV, and 3.12 eV. The PL emissions observed at 381 nm, corresponding to 3.25 eV (Figure 8b), are in close agreement with the band-edge absorption at 3.27 eV (Figure 6b), which was correspondingly observed in the UV–visible spectroscopy. Table 4 lists the PL emissions and fitting PL peaks for ZnO (SZ2) and CuO thin films (SC2).

#### 3.5.2. Photoluminescence of CuO Thin Films

**Figure 9:**
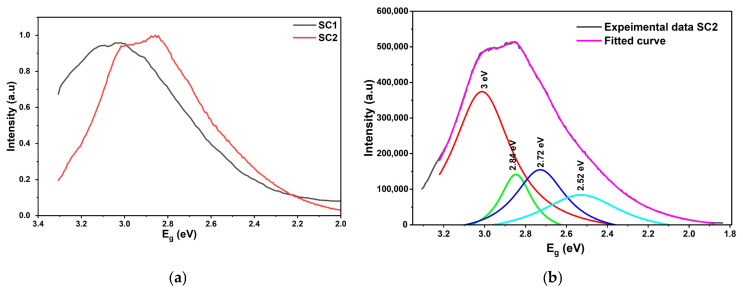
(**a**) Photoluminescence of CuO thin film (SC1 and SC2). (**b**) Peak fit of CuO thin film (SC2).

Similarly, the PL emission spectrum of the CuO thin films, SC1 and SC2, is illustrated in Figure 9a,b. In the unannealed CuO sample, band-edge luminescence is observed at 3 eV (410 nm PL peak wavelength), which closely matches the band-edge absorption at 3.1 eV obtained in the UV–visible spectroscopy, indicating strong consistency between the two characterizations. In the annealed SC2 sample, the PL peak observed at 425 nm, corresponding to the PL emissions at 2.9 eV, also matches well with the absorption edge of 2.89 eV (Figure 6a) estimated from the UV–visible spectroscopy. The study on the defects was carried out using the Lorentzian fit of the PL spectrum for the annealed CuO sample SC2.

The CuO PL peak has been resolved into four emissions at 3 eV (410 nm), 2.85 eV (434 nm), 2.7 eV (456 nm), and 2.54 eV (487 nm). Similar to the ZnO PL peak, Sample SC2 also exhibits asymmetricity, due to the presence of possible Cu interstitials and O_2_ vacancies. Peak fit details of the SZ2 and SC2 thin films are listed in Table 5. There have been several reports of PL peaks at 2.7 eV, which are attributed to the presence of O_2_ vacancies. The broad PL peak is indicative of high levels of defects, creating various traps that lead to sub-bandgap emissions [50].

The presence of localized states and the Urbach tails at 2.89 eV in the tauc plot (Figure 7a) and the broad PL peaks (Figure 9a) are clear evidence of Cu interstitial and oxygen vacancy-related defects in Sample SC2, even after annealing at 300 °C. However, the presence of a weak Urbach tail at 1.25 eV in the corresponding UV-vis spectroscopy (Figure 7b) confirms the presence of the CuO phase.

## 4. Fabrication of Thin Film Heterostructure

### 4.1. Fabrication of CuO/ZnO-Based Thin Film Heterostructure (SD1)

**Figure 10:**
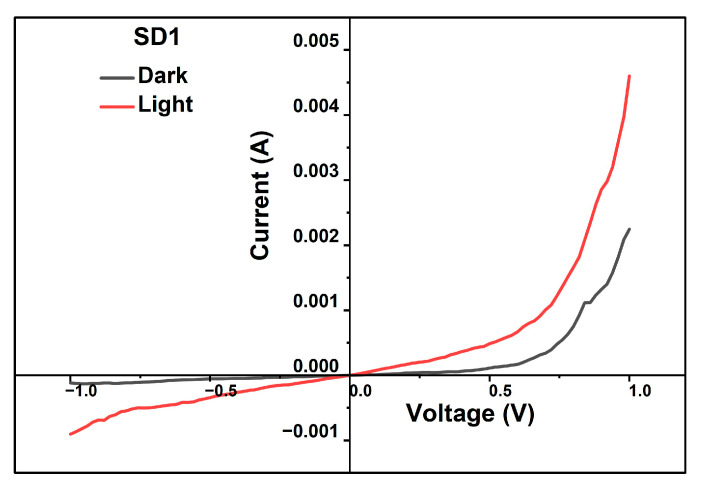
I–V characteristics of device SD1.

In order to evaluate photo currents generated by the CuO/ZnO heterojunction, a thin film hetero structure SD1 was fabricated with the schematic of Al/FTO/CuO/ZnO/Al on an FTO-coated glass substrate (Figure 1a). The details of the thicknesses of CuO and ZnO and their process variations in temperature and the duration of deposition and subsequent annealing are enumerated in Table 2. Photographs of devices SD1 and SD1 with Al patterned contacts at the top and bottom are shown in Figure 1c. Figure 10. shows the current–voltage characteristics of a two-terminal device, SD1. The rectifying behavior is clearly evident from the non-linear increase in the current during forward bias. The rectification ratio of 5.06 (the ratio of forward to reverse bias current) and an ideality factor of 3.37 were obtained for SD1 using Equation (4).

(4) $$n=\frac{q}{K_{B}T}\;\frac{dV}{d(lnI)}$$

*q*—electronic charge (1.6 × 10^−19^ C).

$k_{B}$—Boltzmann constant (1.38 × 10^−23^ J/K).

T—absolute temperature (K).

$I_{d}$—dark current.

$V_{d}$—dark voltage.

A photocurrent of 5 mA was generated at an applied forward voltage of 1 V for an area of 1 × 1 cm^2^. It should be noted that for the given surface area, a photocurrent of 5 mA @ 1 V forward voltage is one of the highest reported in the literature when compared to similar RF-sputtered, electrode-deposited, thermal-evaporated, and chemically synthesized thin film solar cells [2,20,28] and thin film hetero structure [51,52,53], as listed in Table 6.

Estimation of the ideality factor (*n*) indicates the characteristics of the rectifying behavior of the junction. The unity ideality factor represents an ideal junction, typically governed by thermal diffusion process, whereas for *n* > 2 the transport is governed by tunneling process, and for 1 > *n* > 2 the transport is controlled by generation recombination (G-R) process. The rectification ratio and ideality factor calculated from the characteristics deviate from the ideal case due to various factors, such as the presence of defects in the ZnO and CuO active layers. Lattice mismatch between CuO and ZnO, traps in the interface [36], and oxidation on the surface of the metal electrodes could be other possible reasons for the high ideality factors.

Based on our thin film optimizations, it is observed that both the ZnO and CuO thin films exhibit defects. However, our results show that the defects are relatively higher in ZnO when compared to CuO, with the presence of deep Urbach tails. The presence of these defect tails at 3.03 eV and 1.68 eV in the annealed ZnO thin films could be the plausible reason for the high ideality factor of device SD1. The absence of Urbach tails in CuO confirms that there is high absorption in the CuO layer, and hence a high photo-current of 2 mA in the dark and 5 mA under light conditions.

### 4.2. Fabrication of MoS_2_/CuO/ZnO-Based Thin Film Heterostructure

**Figure 11:**
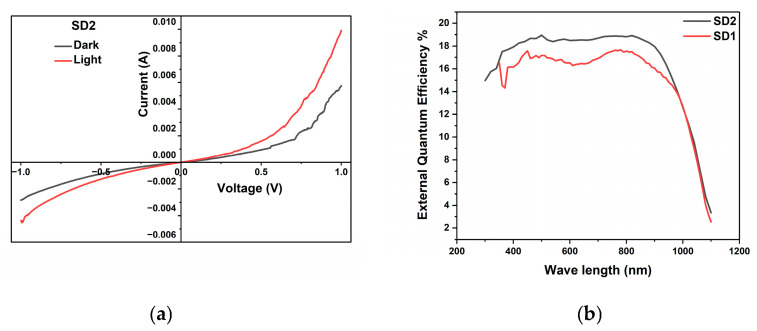
(**a**) I–V characteristics of device SD2. (**b**) EQE measurement of SD1 and SD2.

Figure 11a depicts the I–V characteristics of an Al/FTO/MoS_2_/CuO/ZnO/Al thin film heterostructure. In device SD2, the MoS_2_ layer is sandwiched between the substrate and CuO. All the deposition and annealing parameters are unchanged except for the addition of the MoS_2_ layer, which provides a favorable valence band alignment with CuO, enabling efficient hole extraction and transportation toward the metal contact.

Our previous study on the MoS_2_ layer confirms that the thin film is p-type in nature. Hence, MoS_2_ was added as the hole-transporting layer [54]. Device SD2 exhibits a higher photo current of 5 mA in the dark and 9 mA under light conditions, as compared to device SD1.

Figure 11b shows the external quantum efficiency (EQE) measurement of SD1 and SD2; the absence of measurable V_OC_ and I_SC_ in both devices is attributed to the strong leakage currents of the order mA, which suppress the photo response under standard illumination.

Pseudo-short-circuit currents were calculated for SD1 and SD2 from the I–V reverse bias at −1 V, and determined to be 0.79 mA and 1.55 mA, respectively. In Figure 11b, the overall EQE of SD2 is enhanced from 16% in SD1 to 18% in SD2. The addition of MoS_2_ to the heterostructure enhanced the EQE by 2%, especially due to the possible hole extraction through the MoS_2_ layer. Accordingly, EQE serves as a sensitive diagnostic tool to confirm photoactivity and assess optical/collection losses, despite the lack of resolvable V_OC_ and I_SC_ in the current device configuration.

## 5. Conclusions

In conclusion, ex situ annealing in N_2_ ambient for ZnO thin films and in situ annealing in O_2_ ambient for CuO thin films were systematically studied. In both material systems, XRD analyses revealed that annealing improved the crystalline quality. Raman analyses showed improved ZnO and CuO bonding after annealing. The UV–visible spectroscopy data revealed a bulk-like absorption edge at 3.24 eV for ZnO and 2.89 eV for CuO thin films. In addition to the above, Urbach tails at 3.03 eV and 1.68 eV were observed in ZnO annealed thin film, attributed to the presence of Zn interstitials and oxygen vacancies. Weak Urbach tails were observed only in the as-deposited CuO thin films at 2.8 eV, illustrative of Cu interstitials and oxygen vacancies. Photoluminescence measurement confirms the energy bandgap for both ZnO and CuO. PL studies confirm the presence of defect states in both ZnO and CuO. The presence of PL peak at 400 nm confirms the presence of Zn- and O_2_-related defects, whereas the PL peak at 456 nm confirms the presence of Cu interstitials and O_2_ vacancies. Electrical investigation using Hall measurement revealed a carrier mobility of 35 cm^2^/V-s in CuO Sample SC1, possibly due to high % of Cu interstitials and oxygen vacancies. Post-deposition annealing of CuO resulted in a reduction in Urbach band tail states, which affected the reduction in interstitials and vacancies. Hence, Sample SC2 seems to exhibit the highest carrier mobility of 58 cm^2^/V-s.

Thin film heterostructures were subsequently fabricated based on the optimized deposition parameters of CuO and ZnO. Optimized Al deposition on CuO and Al deposition on ZnO resulted in ohmic bottom and top contacts, respectively. P-CuO/n-ZnO thin film heterosturcture resulted in high photocurrents of 2 mA in the dark and 5 mA under light conditions. However, high leakage currents of the order of mA are a critical concern. Additionally, a hole transport layer of p-MoS_2_ was added to the heterojunction solar cell and was shown to exhibit improved photo currents of 5 mA in dark and 9 mA in light conditions. Lastly, pseudo-short-circuit currents of 790 mA and 1.55 mA were determined for devices SD1 and SD2, respectively, indicating that the addition of a hole transport layer results in improved electrical properties of hetero structure SD2.

## Figures and Tables

**Table 1:** Deposition parameters for zinc oxide and CuO thin films.

Sample	Deposition Temperature	RF Power (W)	Thickness (nm)	Annealing Temperature	Annealing Duration	Annealing Ambient
SZ1 (ZnO)	Room Temperature	60	180	-	-	-
SZ2 (ZnO)	Room Temperature	60	180	350 °C	60 min	N_2_ (Ex situ)
C1 (CuO)	150 °C	65	270	-	-	-
SC2 (CuO)	150 °C	65	270	300 °C	60 min	O_2_ (In situ)

**Table 2:** Device fabrication details.

Device	Active Layer			Hole Transport Layer		Window Layer		Bottom and Top Contact
	Material	Deposition Temperature	Annealing Duration (mins)	Material	Deposition Temperature	Material	Deposition Temperature	
SD1	CuO (180 nm)	150 °C	60	-		ZnO (50 nm)	RT	Al (100 nm)
SD2	CuO (180 nm)	150 °C	60	MoS_2_. (30 nm)	RT	ZnO (50 nm)	RT	Al (100 nm)

**Table 3:** Parameters of ZnO thin films extracted from XRD peak (002).

Sample No	(002) 2*θ* (°)	(002) Peak Intensity (a.u)	FWHM (*β*) (°)	FWHM (*β*) (rad)	Lattice Parameter Spacing (nm)	Grain Size (nm)	Dislocation on Density (nm^−2^)	Lattice Strain
SZ1	34.29 ± 0.00	549.68	0.945 ± 0.00	0.0164	0.26	9.11	0.012	0.032
SZ2	34.45 ± 0.00	1364.09	0.451 ± 0.00	0.0078	0.26	19.27	0.002	0.006

**Table 4:** Parameters of CuO thin films extracted from XRD peak (002).

Type	2*θ* (°)	(002) Peak Intensity (a.u)	FWHM (*β*) (°)	FWHM (*β*) (rad)	Lattice Parameter Spacing (nm)	Grain Size (nm)	Dislocation Density (nm^−2^)	Lattice Strain
SC1	36.81 ± 0.00	399.058	3.86 ± 0.00	0.067	0.24	2.26	0.195	0.2
SC2	35.77 ± 0.00	660.096	0.92 ± 0.00	0.016	0.25	9.48	0.011	0.157

**Table 5:** Photoluminescence peak fit details of SZ2 and SC2 thin films.

Sample	Experimental Peak Value of Energy (eV)	Peak1 (eV)	Peak2 (eV)	Peak3 (eV)	Peak4 (eV)
SZ2	3.1	3.12 ± 0.01	3.1 ± 0.01	3.24 ± 0.01	3.29 ± 0.01
SC2	2.8	3.0 ± 0.01	2.84 ± 0.01	2.72 ± 0.01	2.52 ± 0.01

**Table 6:** Comparison of the CuO/ZnO thin film hetero structure with the literature.

S. No	Year	Deposition Method	Structure	ZnO Thickness (nm)	CuO Thickness (nm)	Photo Current (mA)	Bias Voltage (V)
1	2012 [51]	Electrode deposition	Glass/CuO/ZnO/Al	~1000	~1000	Jsc 1.9/cm^2^	-
2	2015 [52]	VLS-CuO CBD-ZnO	Si/CuO/ZnO	Nano wire	~15	0.00025	−1 to + 1
3	2018 [53]	RF Sputtering	CuO/ZnO/Pt Single cycle	120	400	0.004	−10 to + 10
Comparison of CuO/ZnO thin film solar cell with the literature							
4	2018 [2]	CuO-RF Sputtering ZnO-E Beam	Si/CuO/ZnO	100	200	Jsc 0.44/cm^2^	−1 to + 1
5	2023 [20]	RF Sputtering	TiO_2_/ZnO/CuO	245 354	1654 1487	0.007	0 to 0.02
6	2021 [28]	ZnO-LPCVD CuO-RF Sputtering	ITO/ZnO/CuO/Al	~200	Thin	0.015	−1.5 to + 1.5
7	Our Work	ZnO-RF Sputtering CuO-RF Sputtering	FTO/ZnO/CuO/Al FTO/MoS_2_/CuO/Al	50	180	0.46 9.82	−1 to + 1

## Data Availability

The original contributions presented in the study are included in the article/Supplementary Materials. Further inquiries can be directed to the corresponding author.

## Supplementary Materials

The following supporting information can be downloaded at: https://www.mdpi.com/article/10.3390/ma19040789/s1, Figure S1: Raman spectrum of ZnO thin films SZ1 and SZ2 Figure S2: SEM image of ZnO thin film SZ2.

## Abbreviations

The following abbreviations are used in this manuscript:

RCA	Radio Corporation of America
FTO	Fluorine-Doped Tin Oxide
FWHM	Full Width Half Maximum
Cu	Copper
MoS_2_	Molybdenum disulfide
JCPID	Joint Committee for Powder Diffraction Standards
