# Supplementary material for: Effects of Annealing on the Radio Frequency Sputtered CuO/ZnO Thin Film Heterostructure for Optoelectronic Applications
Source: Materials (Basel). 2026 Feb 18;19(4):789. doi: 10.3390/ma19040789 (PMC12942095; doi:10.3390/ma19040789)
Supplement: Supplementary file 1 [file materials-19-00789-s001.zip › materials-4091839-supplementary.pdf]

Supplementary Information

Effects of_Annealing on the Radio Frequency Sputtered CuO/ZnO Thin Film Heterostructure for Optoelectronic Applications

Sinthamani Sivaprakasam, Sudhakar Bharatan *, Ranjithkumar Mohanam, Sudharsanam Subramaniyam

Department of Electrical and Electronics Engineering, Sri Venkateswara College of Engineering, Sriperumbudur Tk, Kancheepuram Dt, 602117,Tamil Nadu,India.

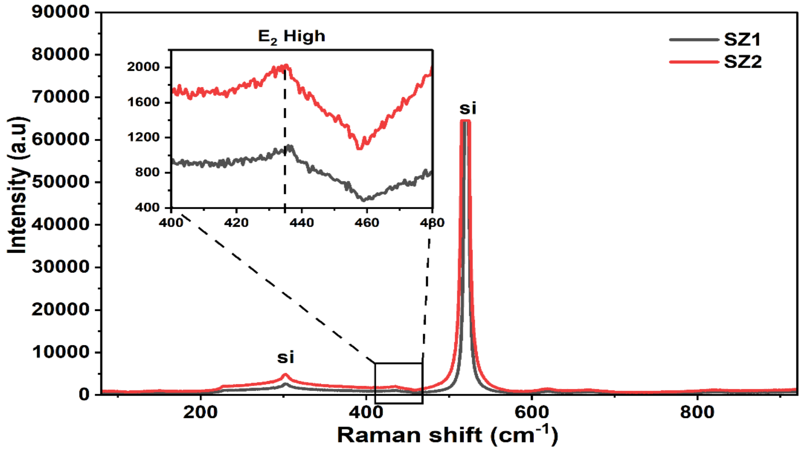

Figure. S1: Raman spectrum of ZnO thin films SZ1 and SZ2

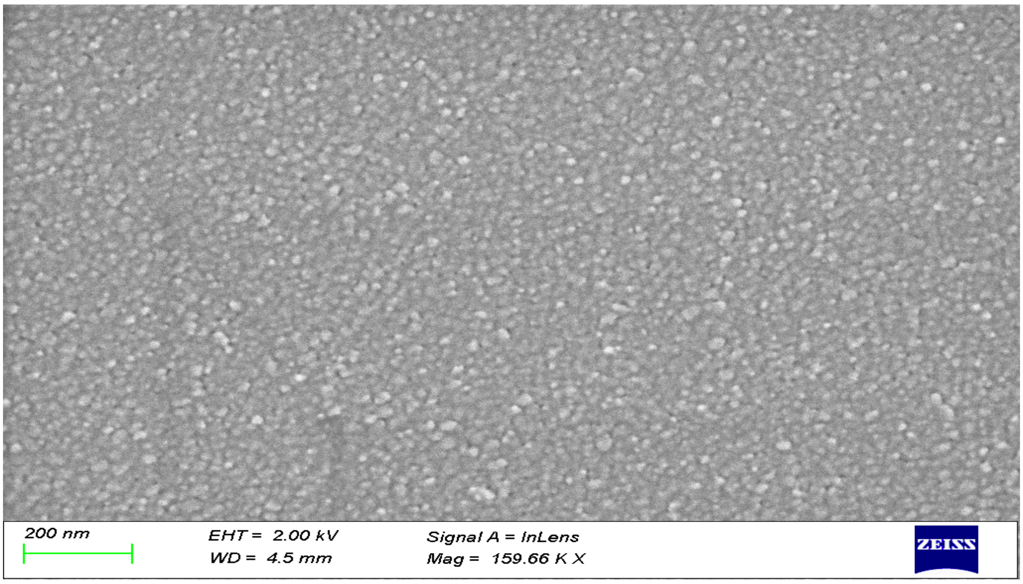

Figure.S2: SEM Image of ZnO thin film SZ2
